# Supplementary material for: Pandemic, Epidemic, Endemic: B Cell Repertoire Analysis Reveals Unique Anti-Viral Responses to SARS-CoV-2, Ebola and Respiratory Syncytial Virus
Source: Front Immunol. 2022 May 3;13:807104. doi: 10.3389/fimmu.2022.807104 (PMC9111746; doi:10.3389/fimmu.2022.807104)
Supplement: Supplementary file 1 [file DataSheet_1.docx]

**SUPPLEMENTARY MATERIALS**

**Supplementary Note. Effect of sequencing depth on estimating repertoire statistics**

We note that in this work there are differences with regards to the sequence counts per sample across the different sample types examined in this analysis (see Supplementary Figure S1). Here we evaluate whether this can impact on the validity of our conclusions, especially when different sample types, owing to difference in sequence counts, introduce different levels of sampling “noise” into the comparisons. We do so by sub-sampling sequences from the repertoire of those CV19 patients and healthy controls with a relatively high (at least 10,000) sequence counts, and then re-calculate the metrics evaluated in the main text. This approach therefore assesses whether the difference in sequence counts impact on the statistical conclusions one can draw from the samples:

1. **Gene usage**

We consider V gene usage and isotype distribution and find that gene frequencies were typically stable across different subsampled sequence counts (Figures i, ii; see below). We acknowledge, however, that this might change if one considers rarer V/D/J genes, as our subsampling analysis suggests that the entire V gene usage profile (i.e. the distribution of frequencies of all V genes examined) is slightly biased at a shallow sequence “depth” (Figure iii, see below).


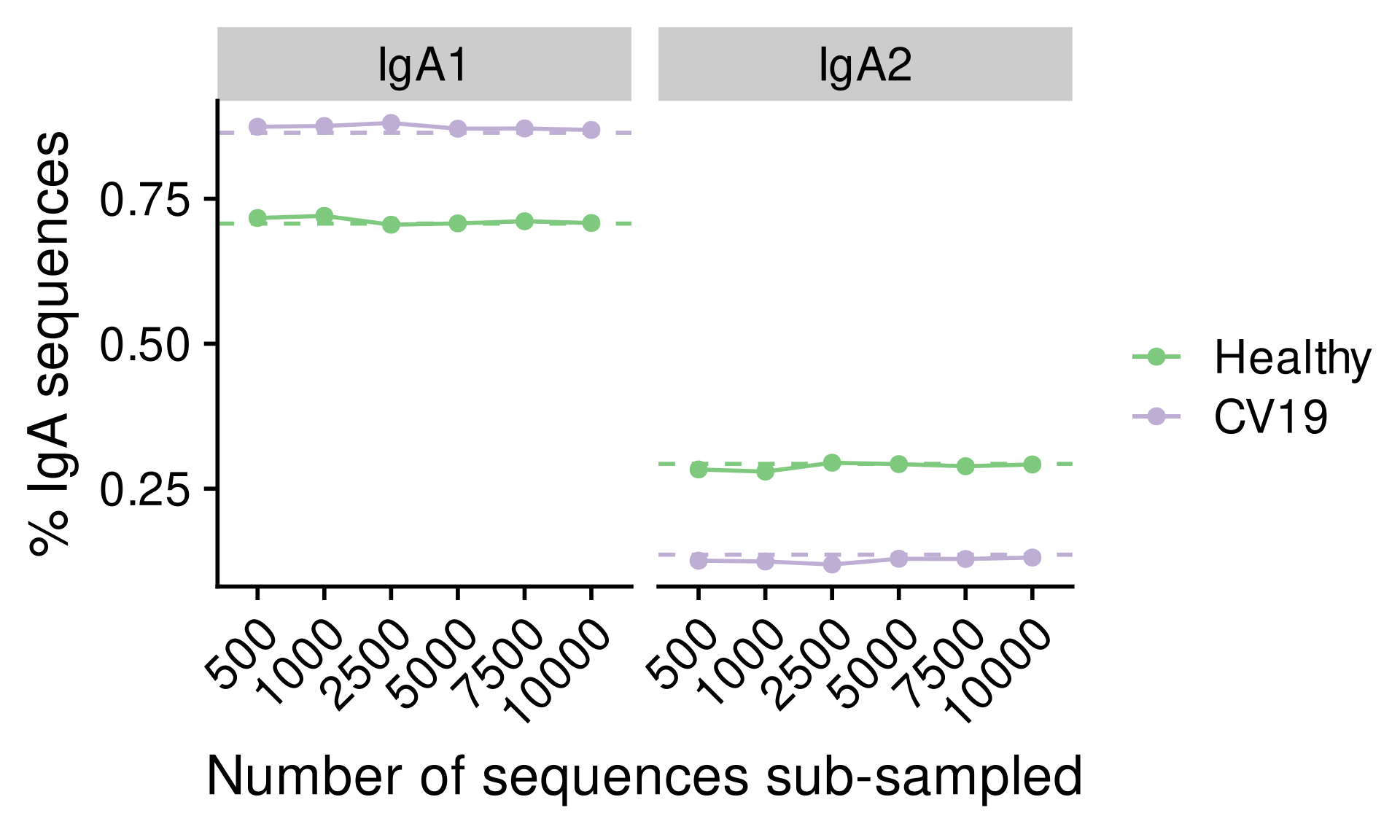

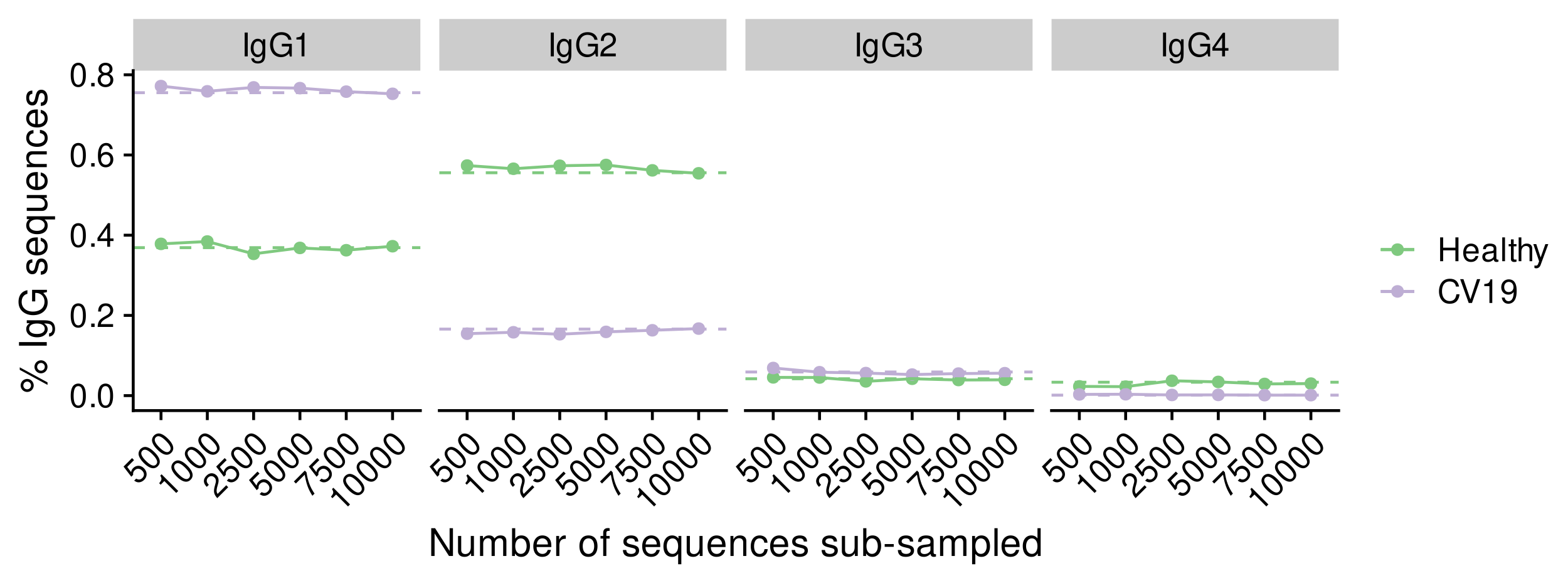


*Fig. i. The effect of sequence counts on isotype usage distribution. IgA (top) and IgG (bottom) subclass usage estimated at different subsampled sequence counts for the Healthy and CV19 samples. The dotted line corresponds to the value calculated over the entire dataset without subsampling.*


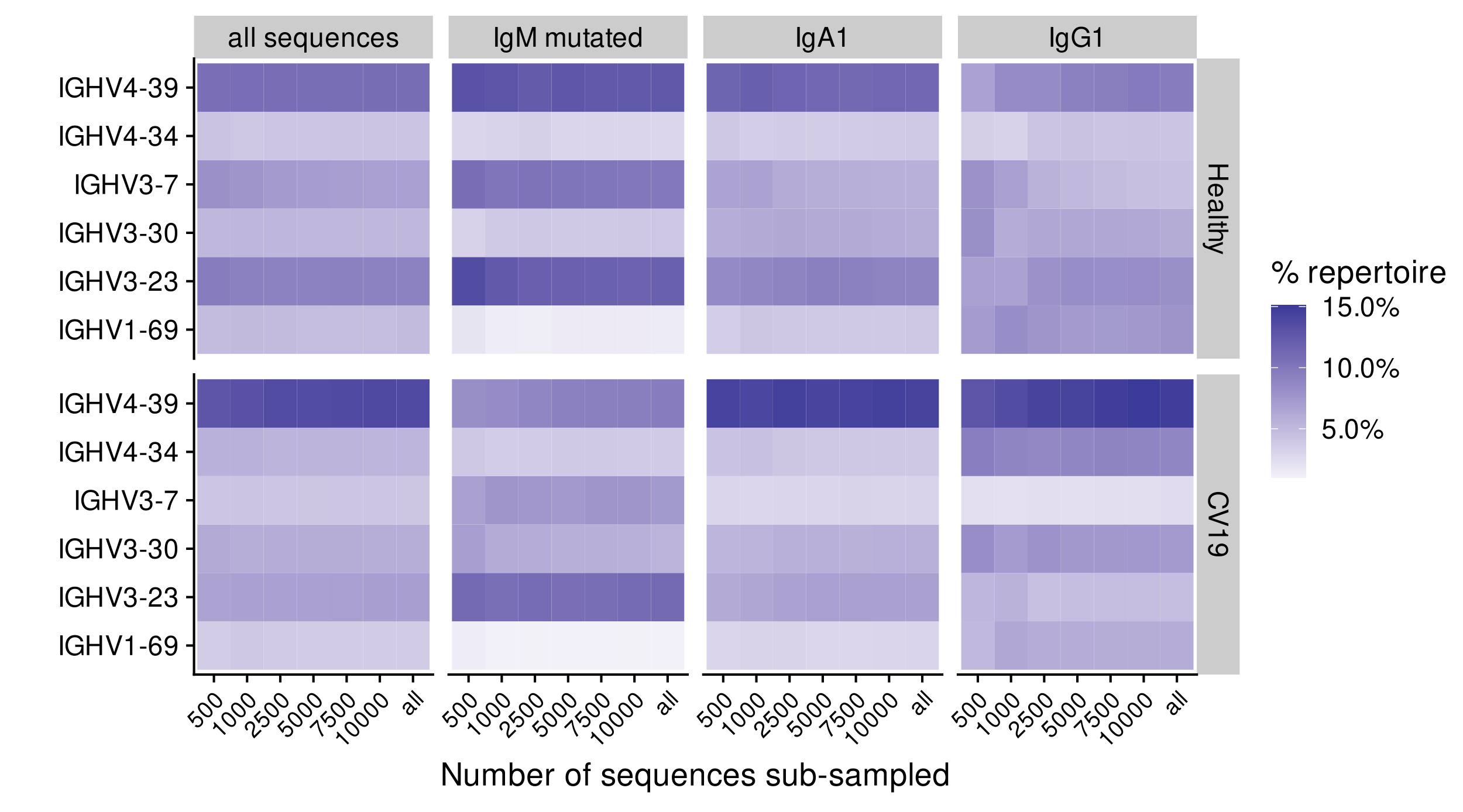


*Fig. ii. The effect of sequence counts on V gene usage estimation. V genes displayed in Figure 2e of the manuscript are depicted here (y-axis) for different sequence counts (x-axis). Gene usage is expressed as the percentage of repertoire, for Healthy and CV19 samples (rows) across different partitions of the repertoire (columns).*


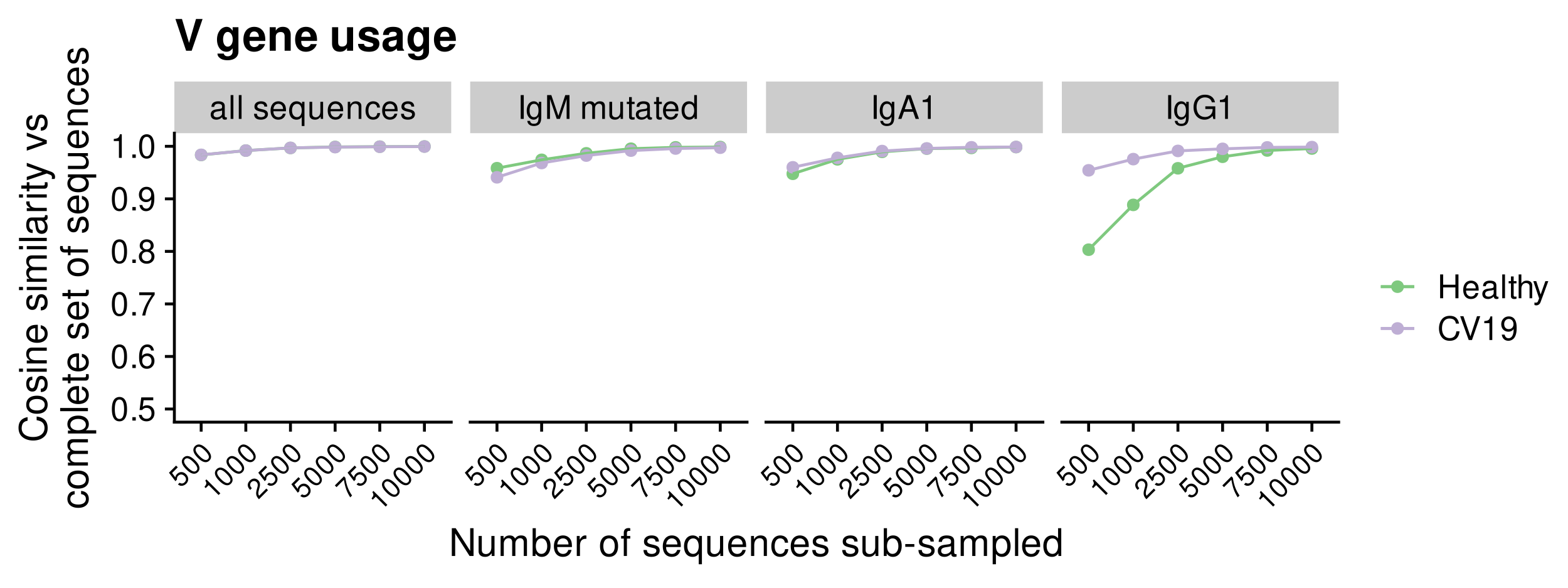


*Fig. iii. The effect of sequence counts on the estimation of V gene usage profile. Usage of all V genes were expressed as the percentage of repertoire and calculated at different subsampled sequence counts, and this frequency vector is compared against the analogous calculation over the entire dataset without subsampling, using cosine similarity (value of 1 = completely identical).*

1. **“Germline likeness”**

Similar to above, we find that the “germline likeness” (i.e. the distribution of distance-from-germline metric evaluated from lineage trees) is stable across sequence counts. Note that even at a very shallow sequence depth, the difference between CV19 and Healthy still stands, albeit with a smaller gap.


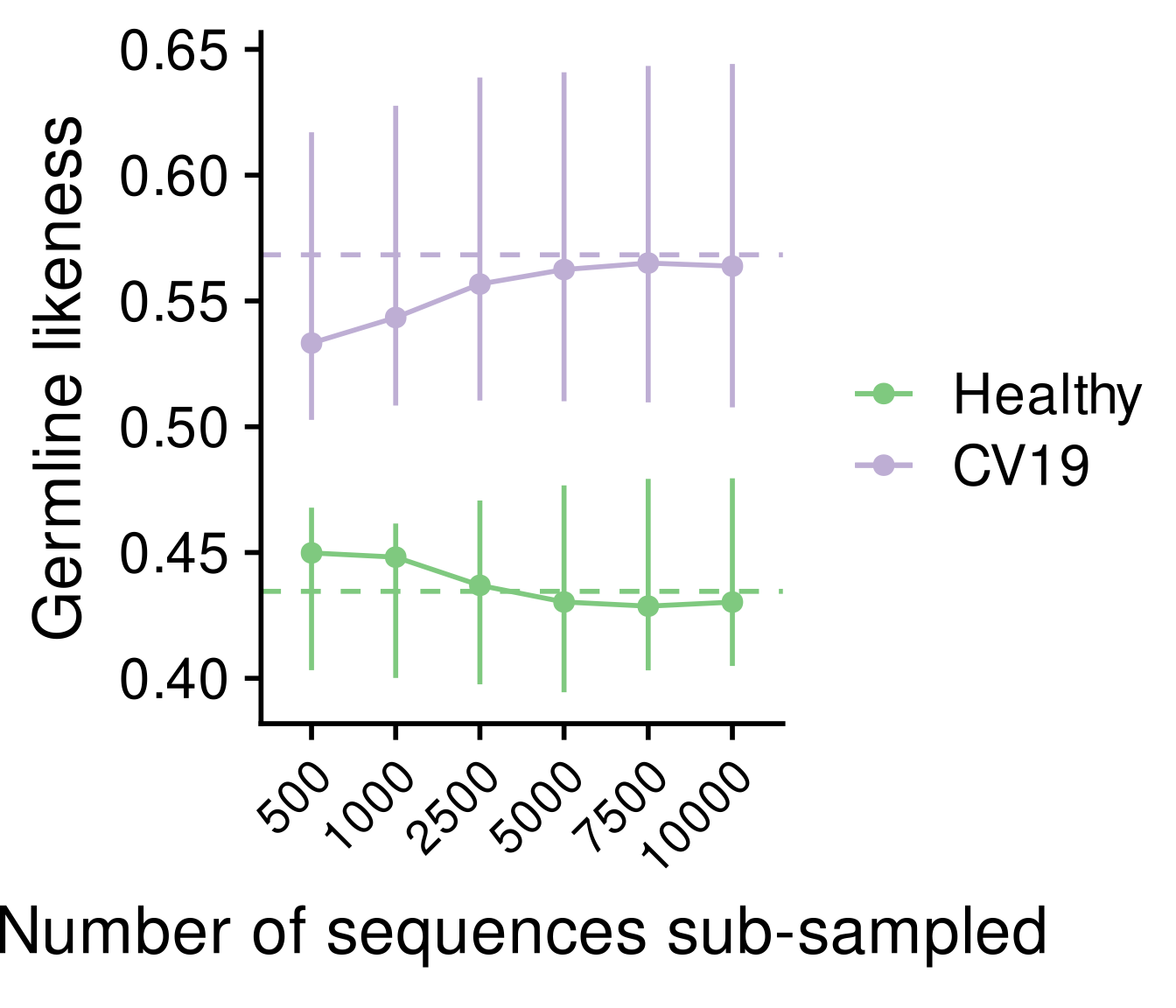


*Fig. iv. The effect of sequence counts on “germline likeness”. The dotted line depicts the value of this metric calculated over the entire dataset without subsampling.*

1. **Clonal diversity and the detection of class-switch recombination events**

As we remarked in the manuscript, the sequence counts impact most on the discovery of clones (that rarer clonotypes are less likely to be observed at a shallow sequence count). We find that when we calculate the Gini coefficient (which quantifies the evenness of the distribution of clone size), this metric is linearly dependent on sequence counts (Fig. v), and for small sequence subsets (e.g. IgG4) it cannot be calculated at a very shallow depth, presumably because clonotypes containing such defined sequence subset do not exist. We however note that at every subsampled sequence depth examined, the directionality of the difference between sample types is preserved (i.e. if the “ground truth” has CV19 higher than Healthy, this difference holds over all subsampled counts examined, albeit at different effect size).


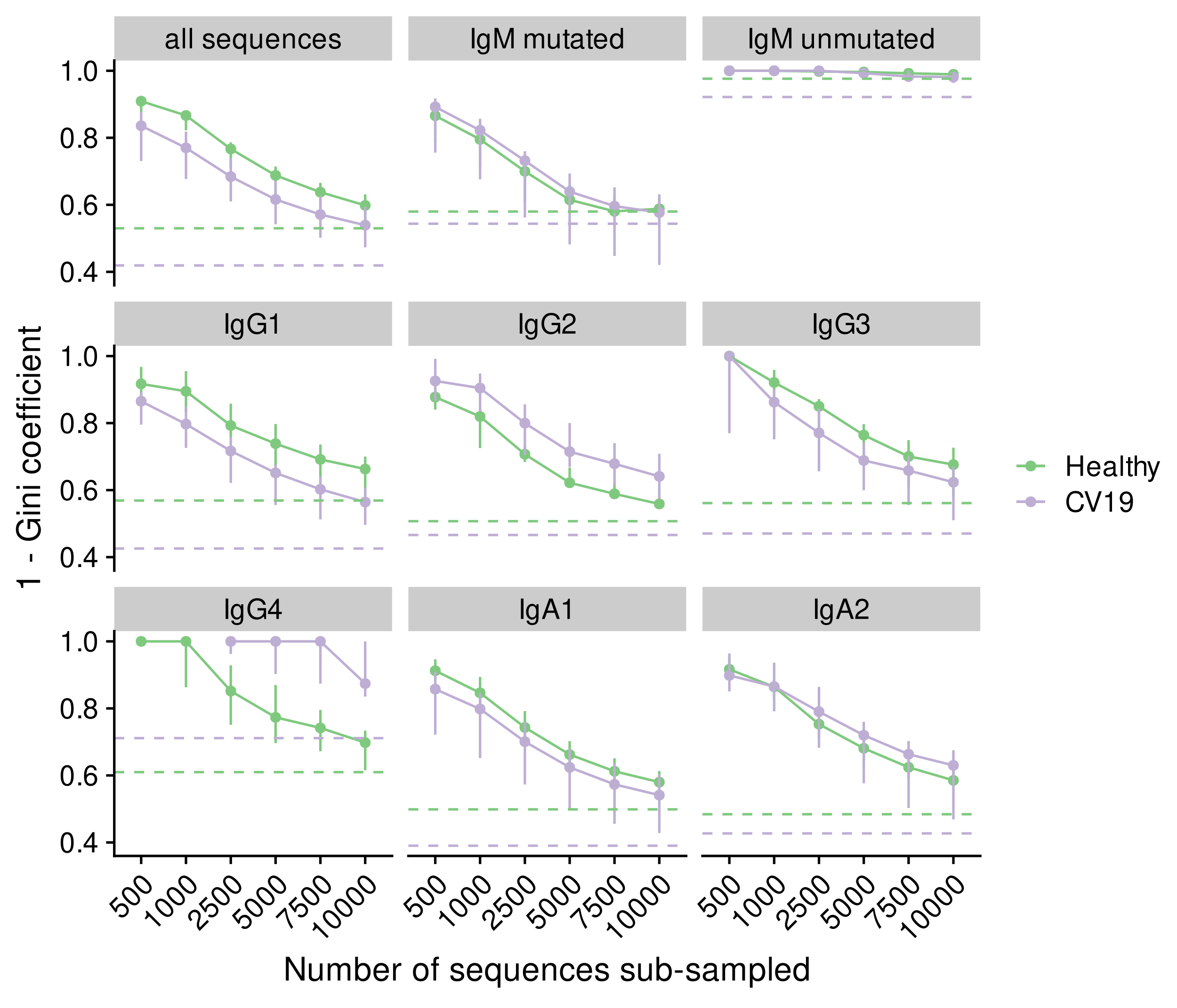


*Fig. v. The effect of sequence counts on estimating clonal diversity. Diversity is expressed as (1 – Gini coefficient), where a lower value indicates an uneven clone size distribution (i.e. expansion of specific clones).*

The impact of sequence counts on clonal diversity also affects the detection of class-switch recombination (CSR) events, since this is defined as pairs of sequences of different isotypes connected within a lineage tree. As expected at a shallow sequence counts little CSR events can be observed (Fig. vi). In our manuscript (Figure 6a) we noted that the comparison Healthy vs CV19 is significant for clones with >10 sequences; here for the smaller clone sizes the subsampled data give a similarly insignificant comparison; for the >10 group we note that across all subsampled depths the difference is not reproduced (Fig. vi, compared with the dotted lines which shows the level calculated over the entire dataset without subsampling).


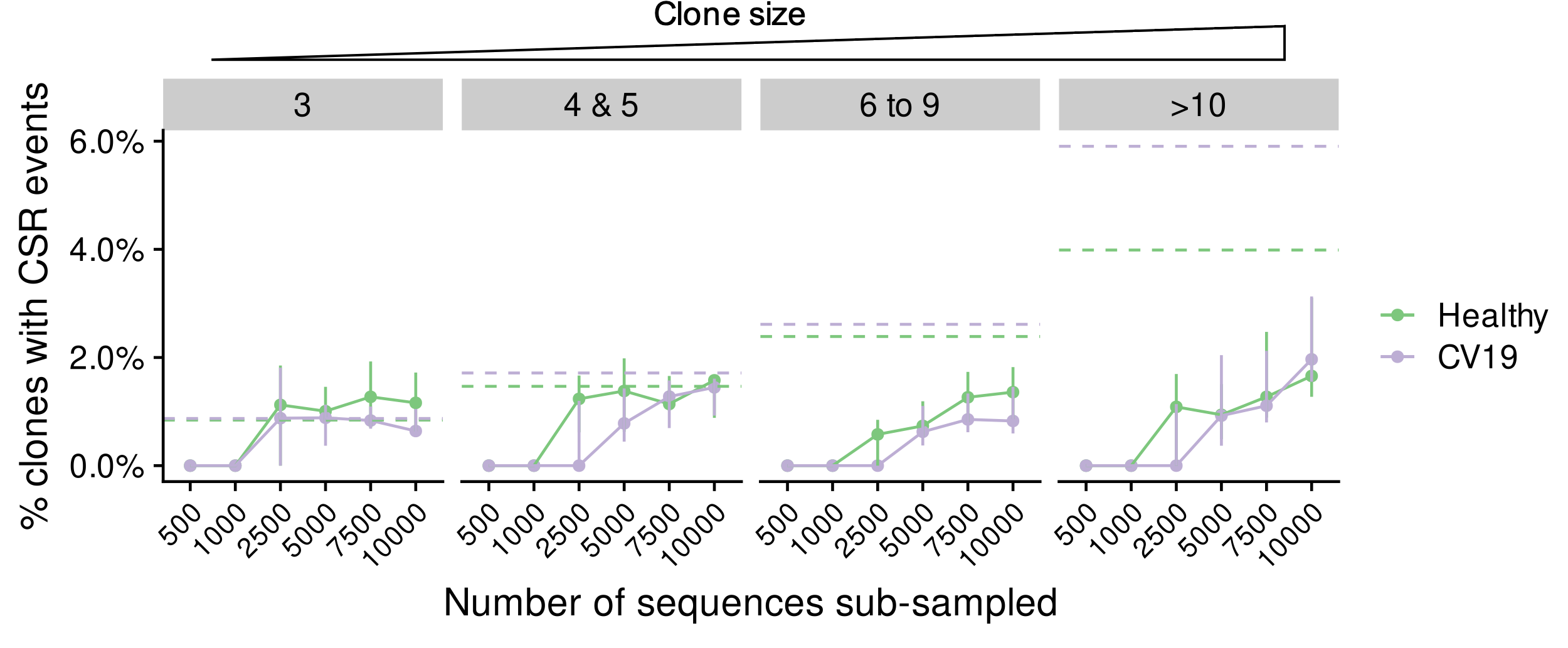


*Fig. vi. The effect of sequence counts on estimating CSR frequency. Clones are grouped by their sizes (columns), and the frequency of CSR events are evaluated across different subsampled sequence depths. The dotted line depicts the levels calculated over the entire dataset without subsampling.*

| **Sample Type** | **Median number of sequences** |
| --- | --- |
| Healthy | 12486.5 |
| CV19 | 38020.5 |
| CV19-Recovered | 3784 |
| EBOV | 5346 |
| RSV-I | 17374 |
| RSV-U | 14686 |
| YFVD28 | 1406 |

*Table ii. Median sequence counts for each sample type investigated in our analysis. The distribution of sequence counts is depicted in Supplementary Figure S1.*

We acknowledge the effect of sequence counts on analysing CSR events. We emphasise, however, that for this subsampling analysis comparing Healthy and CV19, the actual sequence counts are beyond the regime examined in the subsampling (Table ii), indicating that the underestimate of CSR frequencies shown here is likely to be less severe in our actual analysis.

We mainly discussed elevated CSR frequency in the EBOV cohort in comparison to Healthy; we note that the EBOV samples have typically been sequenced to a shallower depth compared to Healthy (Table ii). Extending the arguments put forward above, this could mean that what we observe can be the lower bound of CSR frequencies, i.e. had they been sequenced to a greater depth, more CSR events could be present. For the CV19 samples, we note that the main finding of “early” (i.e. very few mutations) switches from IgM to IgG1 holds across the examined subsampled depths (Fig. vii), although at very shallow depth this could not be estimated, presumably due to the under-sampling of such events at a low sequence counts.


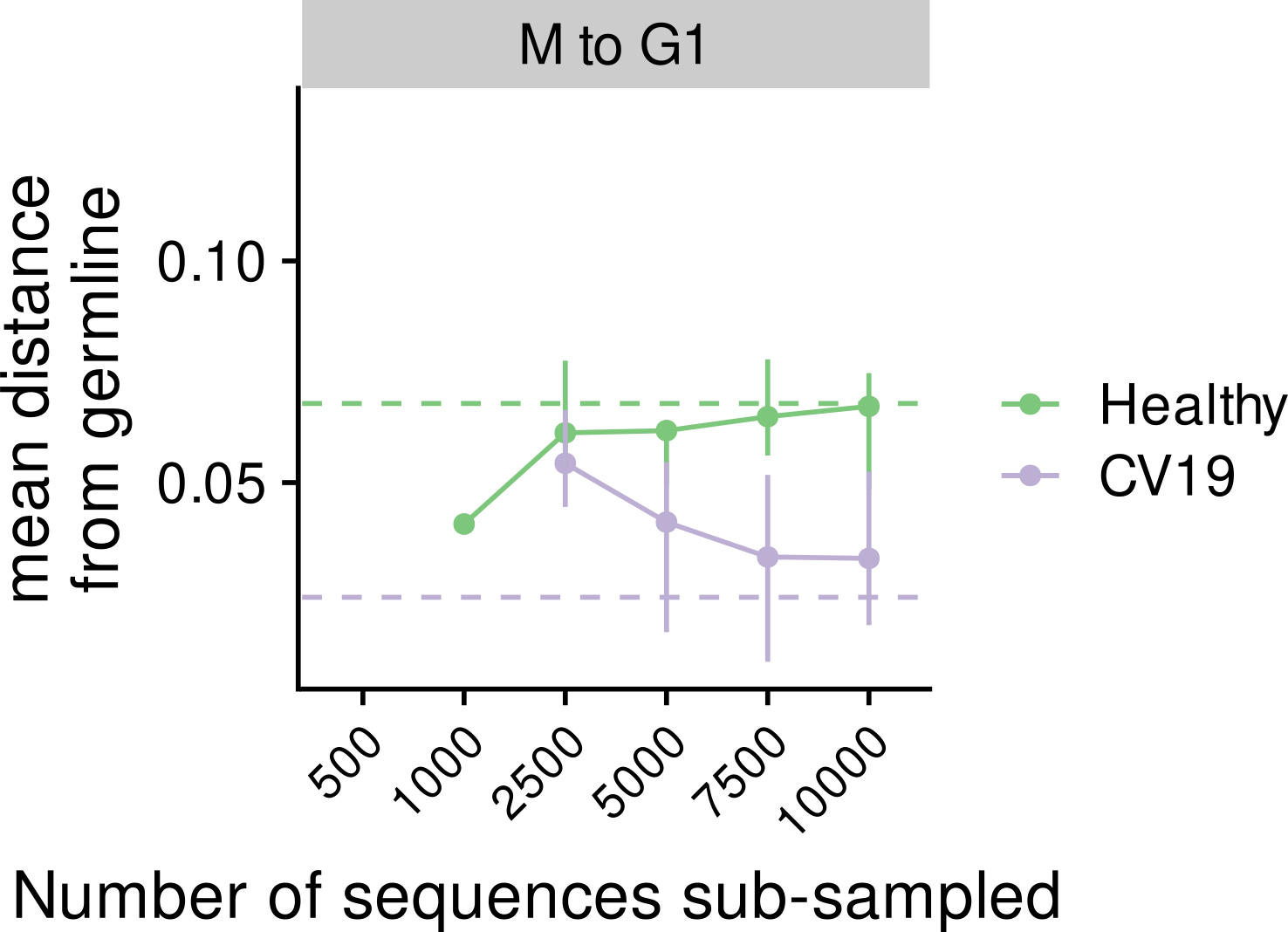


*Fig. vii. The effect of sequence counts on estimating CSR distance-from-germline from IgM to IgG1. Dotted lines indicate the levels estimated from the entire dataset without subsampling.*

In conclusion, the majority of comparisons made in the manuscript are likely to be robust even for fewer sequence counts, with the exception of analyses related to clonal diversity and/or enumeration of CSR events based on the observed lineages. We note that the sequence counts impose a limit on the diversity of the discovered clonotypes and the amount of CSR within clones, especially for samples which have been sequenced to a lower depth.

**Supplementary Methods**

| **Primer** | **Application** | **Sequence (5’-3’)** |
| --- | --- | --- |
| SmartNNN | TSO | AAGCAGUGGTAUCAACGCAGAGUNNNNUNNNNUNNNNUCTT[rG] [rG][rG][rG][rG] |
| Smart20 | PCR1-Forward | CACTCTATCCGACAAGCAGTGGTATCAACGCAG |
| 10XSmart20 | PCR1-Forward | CACTCTATCCGACAAGCAGTCTACACGACGCTCTTCCGATCT |
| IGL-R1 | PCR1-Reverse | ACGGTGCTCCCTTCATGCGTG |
| IGK- R1 | PCR1-Reverse | GTAGTCTGCTTTGCTCAGCGTCAG |
| IGM- R1 | PCR1-Reverse | TGACGTCCTTGGAAGGCAGCAG |
| IGG- R1 | PCR1-Reverse | TCCTGAGGACTGTAGGACAGC |
| IGA- R1 | PCR1-Reverse | CTTCACGTGGCATGTCACGGAC |
| PID-Step | PCR2-Forward | [PID]CACTCTATCCGACAAGCAGT |
| IGL-R2 | PCR2-Reverse | [PID]TTCATGCGTGACCCGGCAGC |
| IGK- R2 | PCR2-Reverse | [PID]TGAGGCTGTAGGTGCTGTCCTTG |
| IGM- R2 | PCR2-Reverse | [PID]CGGGTACTGCTGATGTCAGAG |
| IGG- R2 | PCR2-Reverse | [PID]GACAGCYGGGAAGGTGTGCC |
| IGA- R2 | PCR2-Reverse | [PID]TACAGGTCCCCGGAGGCATC |

Supp. Methods Table 1: Primers used in amplification of B cell repertoire for template switch reverse transcription, gene specific PCR and semi-nested gene specific PCR with Patient Identifier (PID) multiplexing (See Supp. Methods Table 2 for PID sequences).

| **Primer** | **Forward Sequence (5’-3’)** | **Reverse Sequence (5’-3’)** | **Library Usage** |
| --- | --- | --- | --- |
| RSII-PID1 | GGTAGTCATGAGTCGACACTA | CCATCGCGATCTATGCACACG | EBOV, YFV, Healthy |
| RSII-PID2 | GGTAGTATCTATCGTATACGC | CCATCTGCAGTCGAGATACAT | EBOV, YFV, Healthy |
| RSII-PID4 | GGTAGACGTACGCTCGTCATA | CCATCTACAGCGACGTCATCG | EBOV, YFV, Healthy |
| RSII-PID9 | GGTAGTCATGCACGTCTCGCT | CCATCAGTATCACAGTCGCTG | EBOV, YFV, Healthy |
| RSII-PID17 | GGTAGCACGTCACTAGAGCGA | CCATCCAGACGTGACTGATAT | EBOV, YFV, Healthy |
| RSII-PID22 | GGTAGGTGCTGAGCATCAGAC | CCATCTGAGACATACTGAGTG | EBOV, YFV, Healthy |
| RSII-PID23 | GGTAGCACTGATCGATATGCA | CCATCATGTGCACTAGTGTAC | EBOV, YFV, Healthy |
| RSII-PID33 | GGTAGATACAGCACAGATGTG | CCATCGAGTCGTATCGCTCAT | EBOV, YFV, Healthy |
| RSII-PID44 | GGTAGCTCGATACGTGTAGCT | CCATCTGTCAGTAGATGACTC | EBOV, YFV, Healthy |
| RSII-PID45 | GGTAGGTGTCTAGACAGCTGT | CCATCTCGTACGAGATCGACA | EBOV, YFV, Healthy |
| Sequel_PID1 | GGTAGCACATATCAGAGTGCG | CCATCCACATATCAGAGTGCG | CV19, RSV, Healthy |
| Sequel_PID6 | GGTAGCATATATATCAGCTGT | CCATCCATATATATCAGCTGT | CV19, RSV, Healthy |
| Sequel_PID7 | GGTAGTCTGTATCTCTATGTG | CCATCTCTGTATCTCTATGTG | CV19, RSV, Healthy |
| Sequel_PID8 | GGTAGACAGTCGAGCGCTGCG | CCATCACAGTCGAGCGCTGCG | CV19, RSV, Healthy |
| Sequel_PID10 | GGTAGACGCGCTATCTCAGAG | CCATCACGCGCTATCTCAGAG | CV19, RSV, Healthy |
| Sequel_PID12 | GGTAGACACTAGATCGCGTGT | CCATCACACTAGATCGCGTGT | CV19, RSV, Healthy |
| Sequel_PID13 | GGTAGCTCTCGCATACGCGAG | CCATCCTCTCGCATACGCGAG | CV19, RSV, Healthy |
| Sequel_PID15 | GGTAGCGCATGACACGTGTGT | CCATCCGCATGACACGTGTGT | CV19, RSV, Healthy |
| Sequel_PID18 | GGTAGTCACGTGCTCACTGTG | CCATCTCACGTGCTCACTGTG | CV19, RSV, Healthy |
| Sequel_PID20 | GGTAGCACGACACGACGATGT | CCATCCACGACACGACGATGT | CV19, RSV, Healthy |
| Sequel_PID25 | GGTAGCGCGACACGCTCGCGC | CCATCCGCGACACGCTCGCGC | CV19, RSV, Healthy |
| Sequel_PID26 | GGTAGCACAGAGACACGCACA | CCATCCACAGAGACACGCACA | CV19, RSV, Healthy |
| Sequel_PID27 | GGTAGCTCACACTCTCTCACA | CCATCCTCACACTCTCTCACA | CV19, RSV, Healthy |
| Sequel_PID29 | GGTAGTATATATGTCTATAGA | CCATCTATATATGTCTATAGA | CV19, RSV, Healthy |
| Sequel_PID30 | GGTAGTCTCTCTATCGCGCTC | CCATCTCTCTCTATCGCGCTC | CV19, RSV, Healthy |

Supp. Method Table 2: Patient Identifier (PID) multiplexing sequences for the RSII and Sequel PacBio platforms and the libraries generated in which each was used.

**Supplementary Figures**

Figure S1. Relationship between the number of sequences sampled (horizontal axis) and the number of clones clustered (vertical axis) for each repertoire. Each data point represents repertoire from one donor, colour-coded by sample type.

**
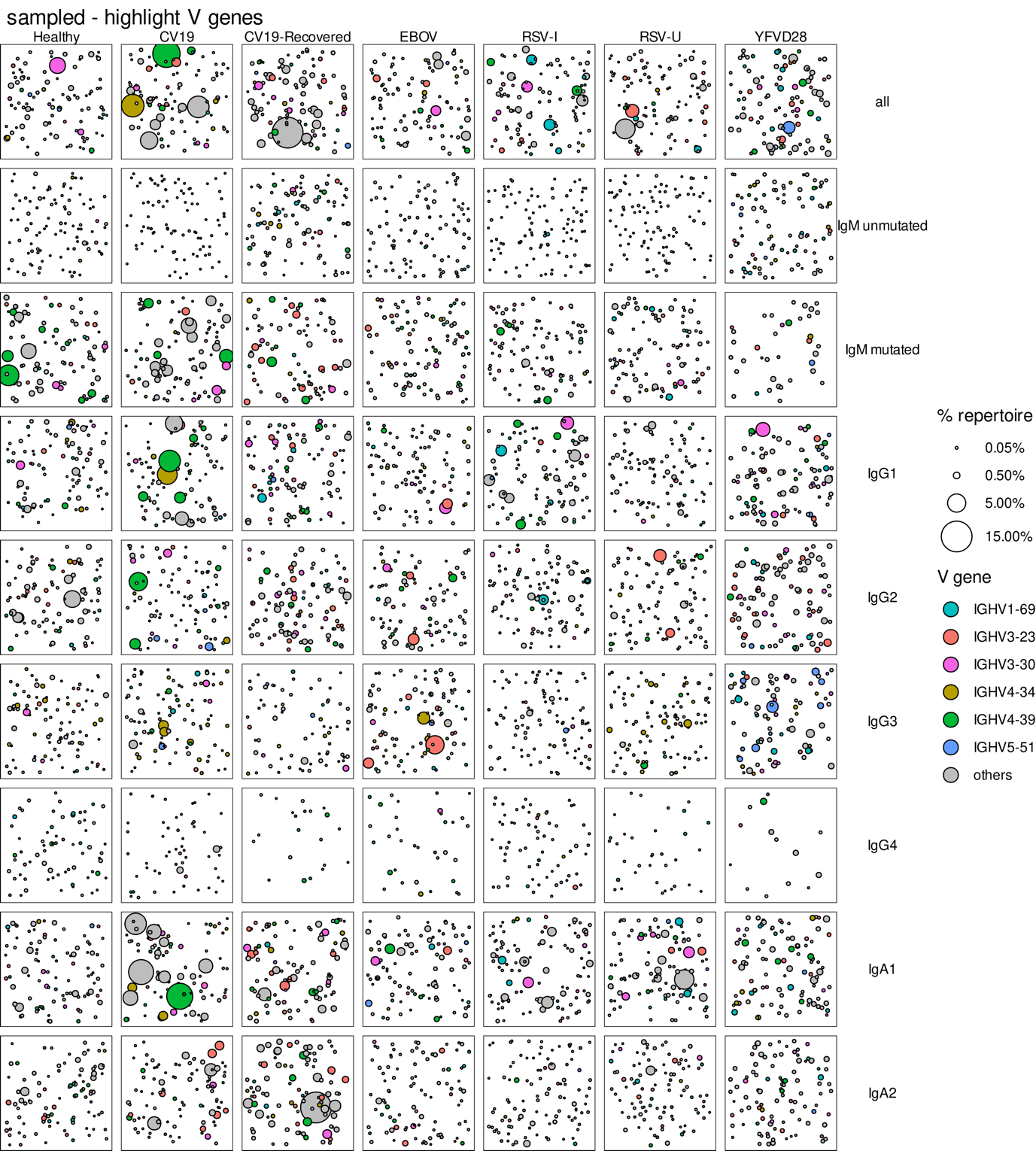
**

Figure S2. Clone size distribution in different cohorts and sequence subsets. Data identical to main text Figure 2C, except that here selected bubbles are colour-coded by V-gene usage discussed in the main text.

**
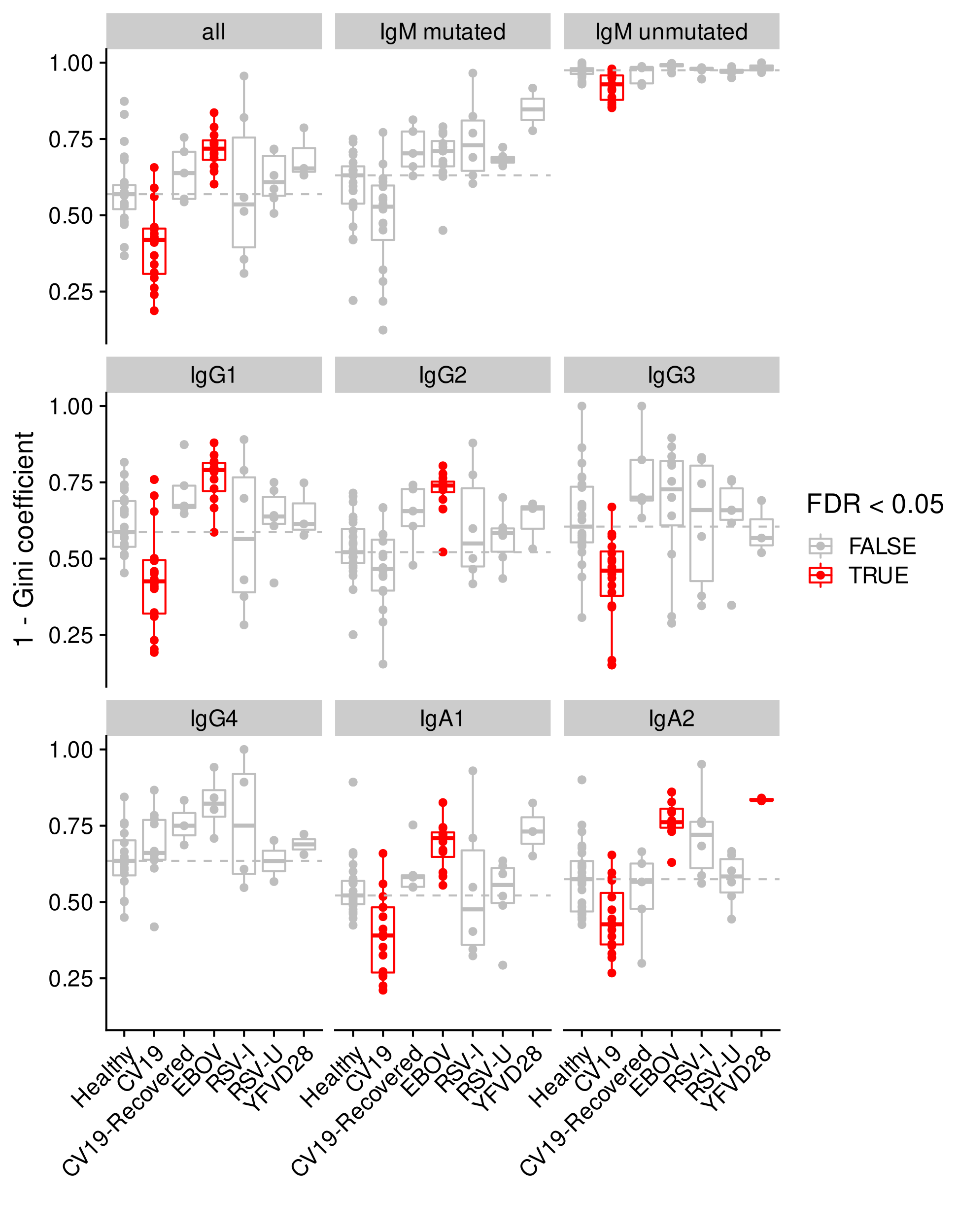
**

Figure S3. Diversity of clone distribution calculated using the formula (1 – Gini coefficient calculated on the clone size distribution). A larger value (closer to 1) indicates polyclonality and a smaller value (closer to 0) indicates monoclonality. Statistical significance was evaluated using a one-way ANOVA and Dunnett post-hoc comparison against the Healthy cohort; those with a false discovery rate (FDR, corrected using the Benjamini-Hochberg method) < 0.05 were highlighted in red. Dashed line indicates the median diversity in the Healthy cohort as a reference. Selected panels are included as main text Figure 2D.


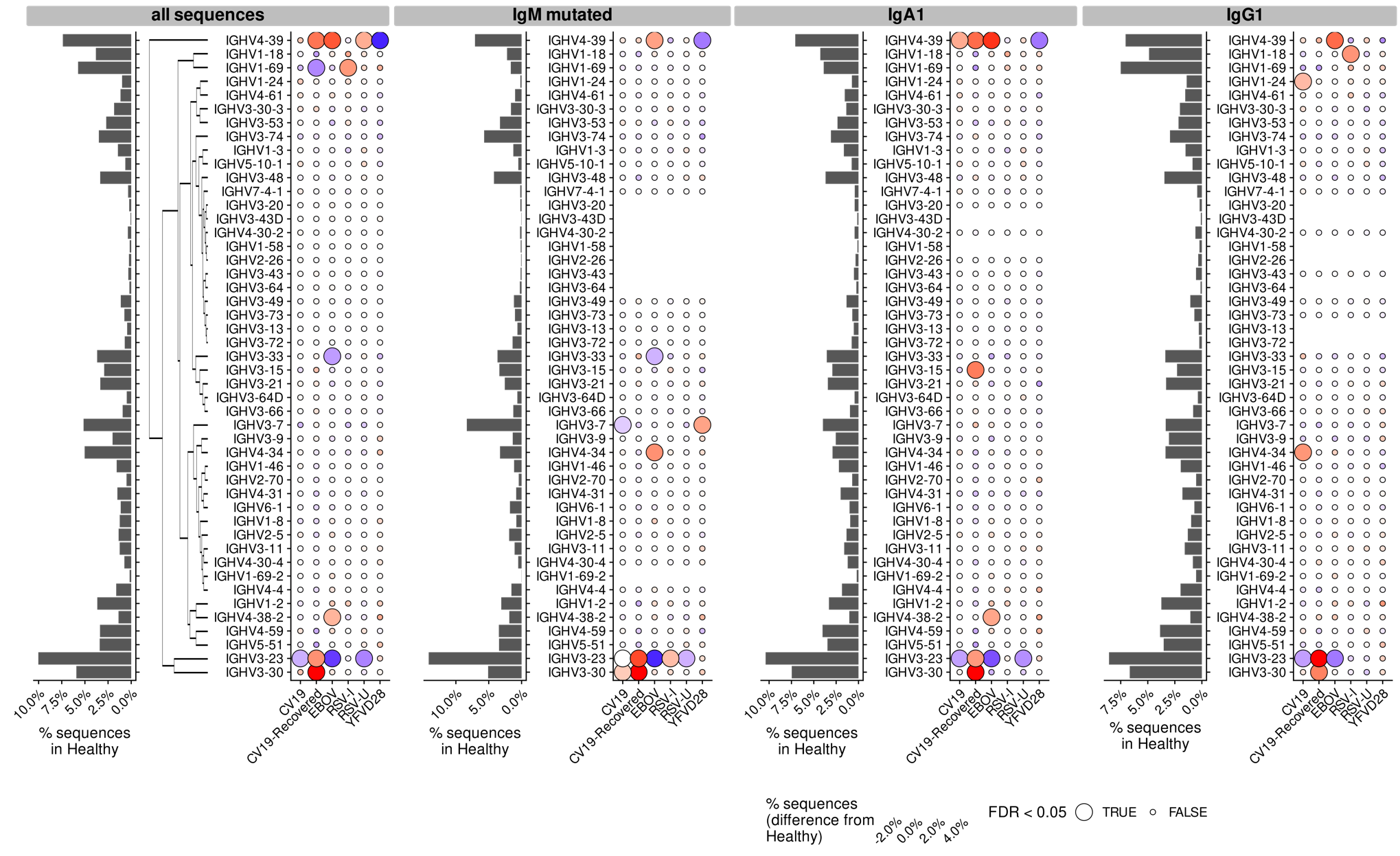


Figure S4. Usage of V genes (vertical axis) in Healthy (left, bar charts) and disease states (dot plots with colours and sizes representing difference and statistical significance in comparison to Healthy). Ordering of V genes is determined by hierarchical clustering of the V gene usage data from all sequences.


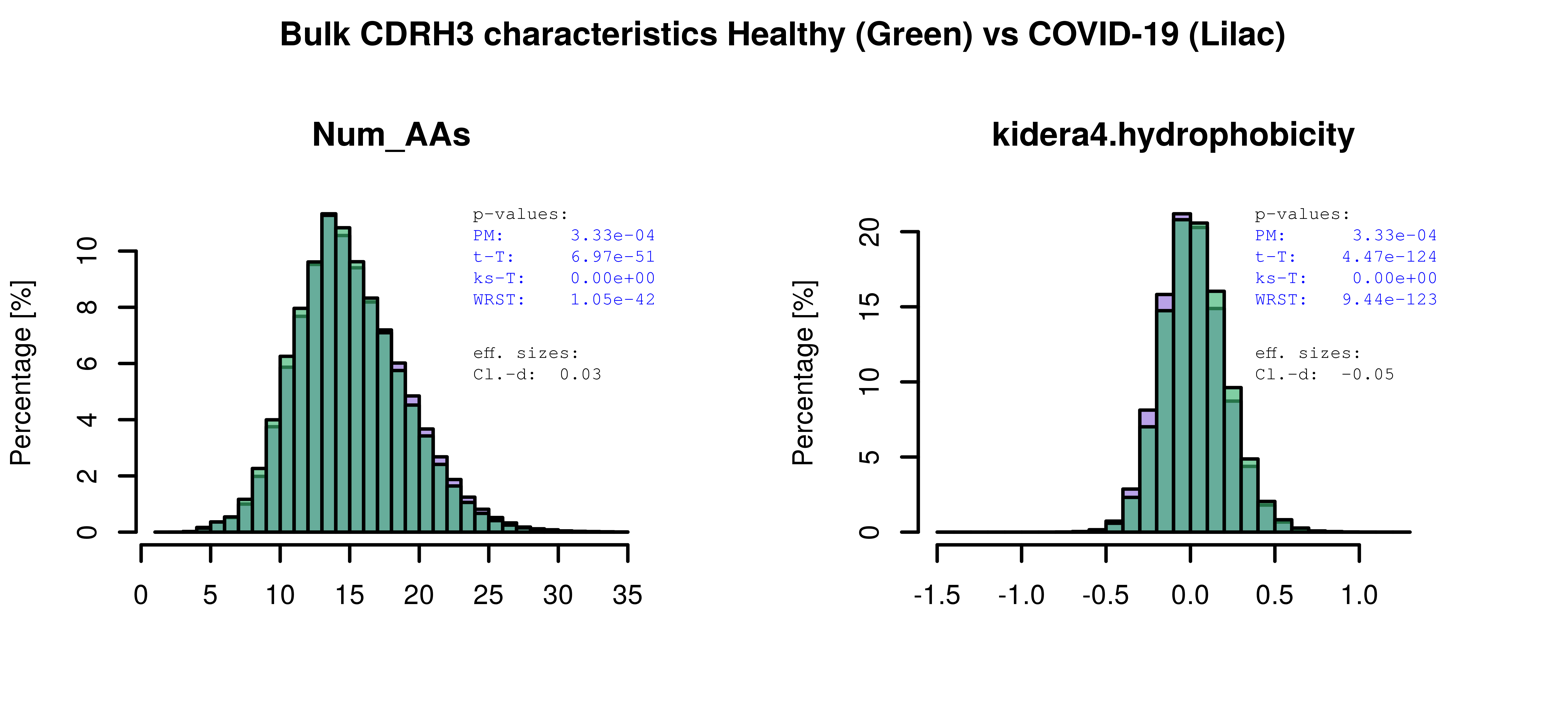


Figure S5. Histograms showing length (number of amino acids, left) and kidera factor 4 (hydrophobicity, right) distributions for CDR3 sequences from Healthy (green) and CV19 (lilac) repertoires. Here all sequences are considered without partitioning by isotypes and mutational level. Statistical comparisons and effect size (Cliff’s delta) calculations were computed on the BRepertoire server (Margreitter et al, 2018).


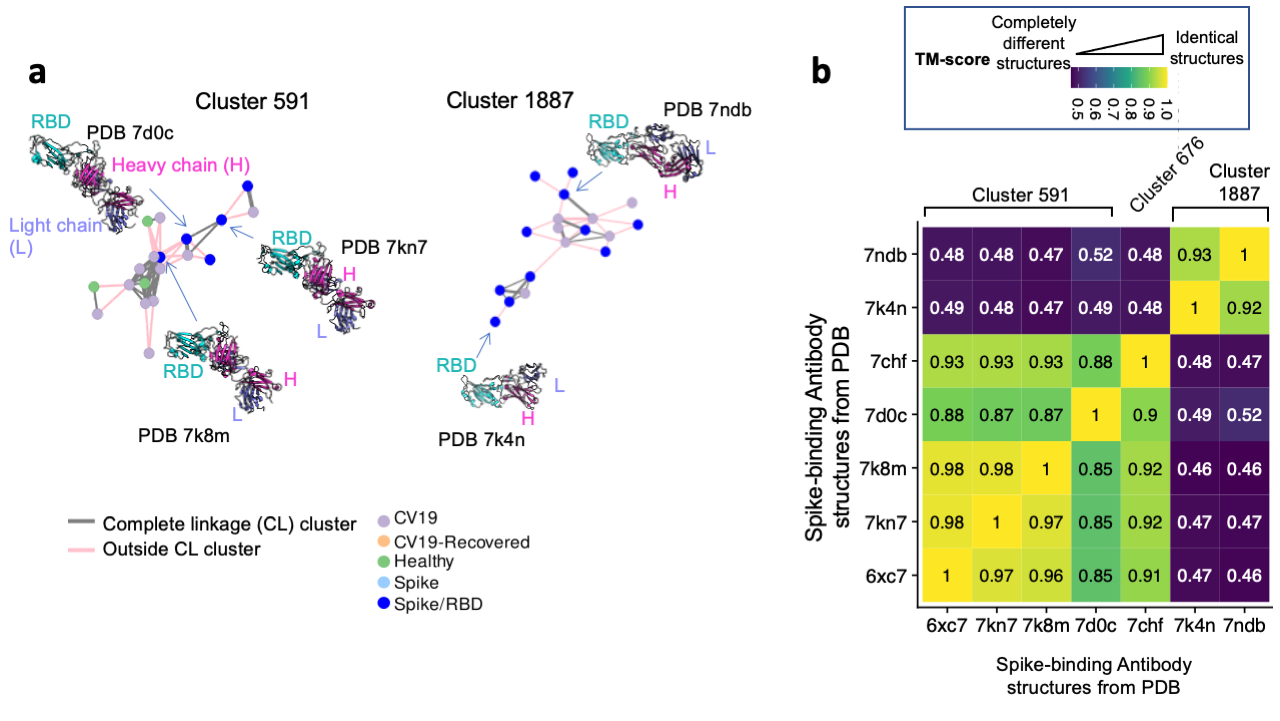


Figure S6. Visualising convergent networks comprising binders with known structural information. (**a**) Here two of the convergent network clusters depicted in Figure 3b are depicted; these clusters have more than one antibody-antigen complex structure deposited in the Protein Data Bank (PDB). Edges are drawn according to the criteria described in Methods and illustrated in Figure 3a, and coloured according to whether the same connections survive under complete linkage (CL) clustering (grey), or would have been removed under CL clustering (pink). Antibody-antigen structures are visualised in the same orientation with respect to the SARS-CoV-2 spike receptor binding domain (RBD). (**b**) Quantification of the similarity in antigen-antibody interactions of all binders depicted in Figure 3b where antigen-antibody structures are available. Pairs of structures are evaluated for their similarity using TM-score. The clusters each binder belongs are indicated in the margin. Note that binders belonging to the same convergent cluster have highly identical structures; some of these would have been classified separately using CL clustering (e.g. the PDB structure 7k8m would have been grouped differently from 7kn7 and 7d0c).

Figure S7. (**a,b**) Summary of convergent CDR3 sequence clusters for (**a**) EBOV and (**b**) RSV repertoire sequences. The construction of convergent CDR3 network is identical to what described in main text Figure 3A. Here shows the size and make-up (top, bar charts) of convergent sequence clusters with at least 10 sequences, as well as the V gene (middle, dark rectangle indicates this gene is used in the given convergent cluster) and J gene (bottom) usage. (**c**) Convergent clusters for RSV repertoire with known binders of the fusion glycoprotein (‘F’). Colour scheme follows that of panel B. The V/J gene usage and CDRH3 amino acid sequences are shown.

Figure S8. Convergent sequence clusters constructed considering CV19, EBOV and RSV repertoire and known binders altogether. This was performed to identify convergent clusters unique to a disease condition or universal across different conditions. Identical procedures as depicted in main text Figure 3A were followed. n = 64 clusters with more than 10 sequences were obtained. Panels **a, c, d, e** are identical to what shown in main text Figure 3C, depicting the breakdown (panel **a**), V gene usage (**c**) J gene usage (**d**) and number of donors represented (**e**) in each cluster. Panel B is an alternative representation of data in (**a**) with dots representing presence of different sequence types (vertical axis). This is provided to better highlight clusters shared across different sample types.


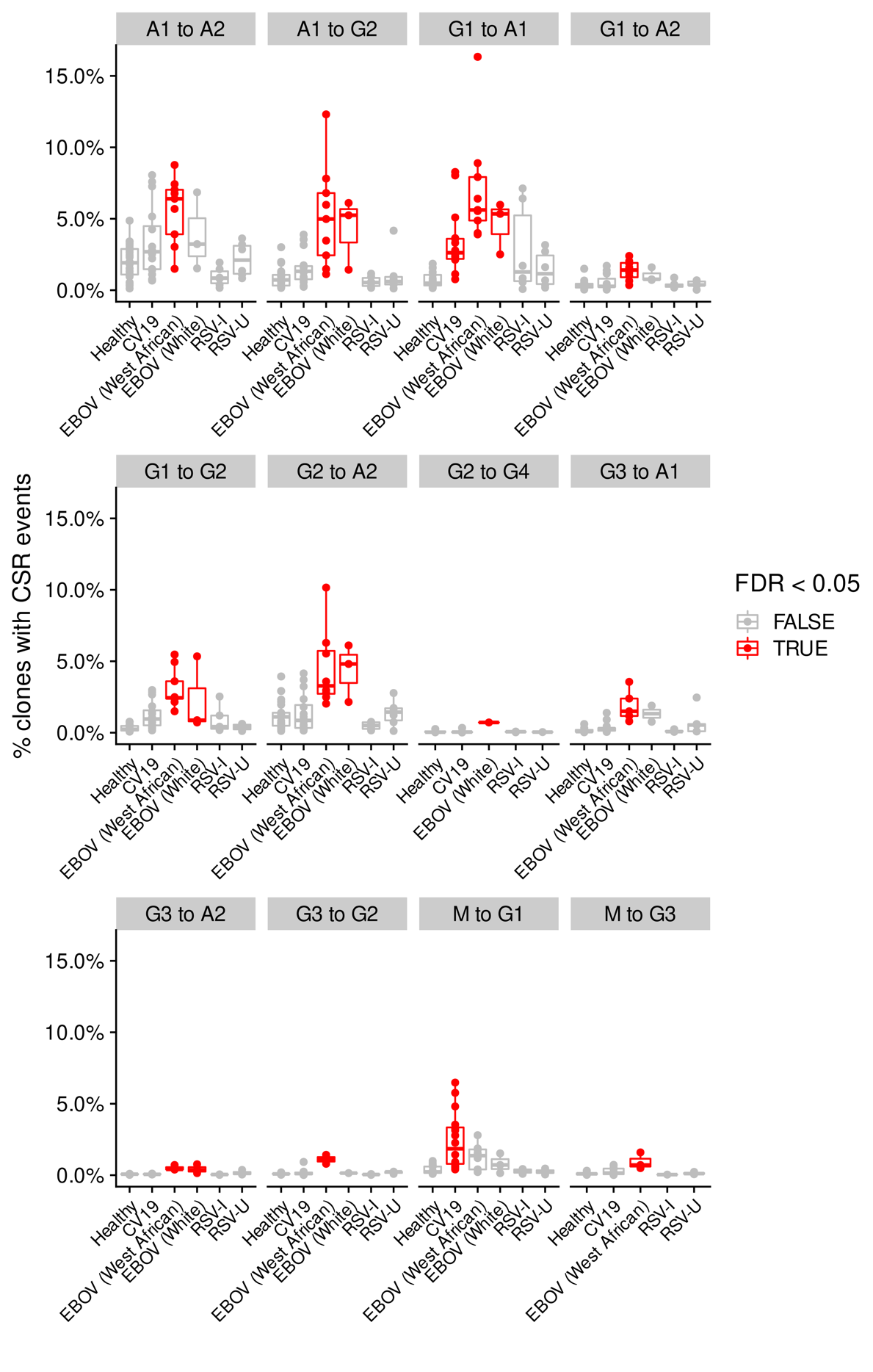


Figure S9. CSR Frequency (proportion of clones with CSR events) comparison across Healthy and disease states. Disease states with significant difference from Healthy (One-way ANOVA followed by Dunnett post-hoc comparison; false discovery rate [FDR] computed using the Benjamini-Hochberg method, FDR < 0.05) are highlighted in red. Data identical to that shown in main text Figure 6C except that here all CSR combinations with at least one comparisons with FDR < 0.05 are represented.

**Supplementary Tables**

Table S1 Donor characteristics.

Table S2 Clone distribution.

Table S3 Gene usage.

Table S4 CDR3 characteristics.

Table S5 Known antibody targeting SARS-CoV-2 proteins.

Table S6 Sequences present in convergent binder networks.

Table S7 Germline likeness of repertoires.

Table S8 Frequency and distance-from-germline analysis of CSR events.
