# Supplementary material for: Pandemic, Epidemic, Endemic: B Cell Repertoire Analysis Reveals Unique Anti-Viral Responses to SARS-CoV-2, Ebola and Respiratory Syncytial Virus
Source: Front Immunol. 2022 May 3;13:807104. doi: 10.3389/fimmu.2022.807104 (PMC9111746; doi:10.3389/fimmu.2022.807104)
Supplement: Supplementary file 2 [file Table_1.docx]

| **Sample** | **Age** | **Gender** | **Ethnicity** | **COVID-19 Severity Score (out of 6)** | **Days since symptom onset** |
| --- | --- | --- | --- | --- | --- |
| **Healthy  (n = 24)** | Median 29.5 (Range 23 - 76)  ≤50 years old: 15/24 (62.5%)  ≥60 years old: 9/24 (37.5%) | Female: 7/24 (29.2%)  Male: 5/24 (20.8%)  Unknown: 12/24 (50%) | White: 12/24 (50%)  Unknown: 12/24 (50%) |  |  |
|  |  |  |  |  |  |
| **COVID-19 (n = 16)** | Median 50.5 (Range 28 - 87)  ≤50: 8/16 (50%)  50-60: 3/16 (18.75%)  ≥60: 5/16 (31.25%) | Female: 7/16 (43.75%)  Male: 9/16 (56.25%) | White: 13/16 (81.25%)  South East Asian:   1/16 (6.25%)  Indian Subcontinent:  2/16 (12.5%) | Median 3 (Range 1 - 5) | Median 8 (Range 1 – 35) |
|  |  |  |  |  |  |
| **COVID-19 Recovered (n = 5)** | Median 50 (Range 28 - 87)  ≤50: 3/5 (60%)  ≥60: 2/5 (40%) | Female: 3/5 (60%)  Male: 2/5 (40%) | White: 4/5 (80%)  Indian Subcontinent:  1/5 (20%) |  |  |
|  |  |  |  |  |  |
| **RSV Infected  (n = 6)** | Young: 3/6 (50%)  Older: 3/6 (50%) |  |  |  |  |
|  |  |  |  |  |  |
| **RSV Uninfected (n = 6)** | Young: 3/6 (50%)  Older: 3/6 (50%) |  |  |  |  |
|  |  |  |  |  |  |
| **Ebola  (n = 12)** | Young: 3/12 (50%)  Unknown: 9/12 (50%) | Female: 1/12 (8.3%)  Male: 2/12 (16.7%)  Unknown: 9/12 (75%) | White: 3/12 (25%)  West African:  9/12 (75%) |  |  |
|  |  |  |  |  |  |
| **YFV D28 (n = 3)** | Median 28 (Range 27 - 28)  Young: 3/3 (100%) | Female: 1/3 (33.3%)  Male: 2/3 (66.7%) | White: 3/3 (100%) |  |  |

Table 1. Donor characteristics. See Supplementary Table S1 for a detailed summary of metadata per donor.
