# Supplementary material for: Pandemic, Epidemic, Endemic: B Cell Repertoire Analysis Reveals Unique Anti-Viral Responses to SARS-CoV-2, Ebola and Respiratory Syncytial Virus
Source: Front Immunol. 2022 May 3;13:807104. doi: 10.3389/fimmu.2022.807104 (PMC9111746; doi:10.3389/fimmu.2022.807104)
Supplement: Supplementary file 3 [file Table_2.docx]

| **Patient** | **Gender** | **Ethnicity** | **Age** | **Severity score** | **Comorbidities** | **Days since symptom onset** | **CRP**  **Levels (mg/L)** | **Oxygen required?** | **MADU?** | **ICU?** | **Total number of sequences** | | | | |
| --- | --- | --- | --- | --- | --- | --- | --- | --- | --- | --- | --- | --- | --- | --- | --- |
|  |  |  |  |  |  |  |  |  |  |  | **D0** | D2 | D9 | D22 | CVR |
| CV221 | M | South East Asian | 29 | Unknown | Brugada syndrome | 9 | 146 | Yes | Y | N | 10558 | 7776 | - | - | - |
| CV222 | M | White | 67 | 3 | High cholesterol & gastro-oesophageal reflux disease (GORD) | 35 | 108 | No | N | N | 6371 | 5311 | - | - | 2066 |
| CV223 | M | Indian Subcontinent | 41 | 3 | Type 2 diabetes | 14 | 229 | Yes | Y | N | 2382 | - | - | - | - |
| CV224 | F | White | 64 | 5 | None given | Unknown | 112 | Unknown | Unknown | Unknown | 239 | 85 | - | - | 498 |
| CV225 | M | Indian Subcontinent | 36 | 3 | None | 8 | 327 | Yes | Y | N | 13415 | 5810 | - | - | 527 |
| CV226 | M | White | 55 | 3 | High cholesterol, ischemic heart disease & depression | 8 | 198 | Yes | Y | N | 7050 | 4840 | - | - | - |
| CV227 | M | White | 56 | 3 | Gout, GORD, pulmonary embolism & rheumatoid arthritis | 14 | 153 | Yes | Y | N | 6552 | 2316 | 4860 | - | - |
| CV233 | F | White | 87 | Unknown | Hypertension, congestive cardiac failure, hyponatraemia & atrial fibrillation | 9 | 210 | No | N | N | 5928 | - | - | - | - |
| CV234 | M | White | 80 | 3 | Ischemic heart disease, chronic kidney disease, abdominal aortic aneurysm & leg ulcers | 5 | 152 | No | N | N | 9196 | 3384 | - | - | - |
| CV239 | F | White | 51 | 2 | Hypertension, type 2 diabetes & pulmonary embolism | 1 | 85 | Yes | N | N | 9721 | - | - | - | - |
| CV247 | F | White | 28 | Unknown | Type 2 diabetes, idiopathic intracranial hypertension , adrenal insufficiency, non-alcoholic fatty liver disease , myopathy, osteopenia, gastroparesis. | 1 | 27 | Unknown | Unknown | Unknown | 11943 | 4774 | 5581 | 7457 | 4367 |
| CV256 | F | White | 38 | 1 | Hypothyroid, hepatitis, nephrotic syndrome | 7 | 32 | No | N | N | 10038 | - | - | - | - |
| CV258 | M | White | 61 | 3 | None | 7 | 299 | Yes | Y | Y | 9661 | - | - | - | - |
| CV293 | F | White | 50 | 2 | Asymptomatic. Appendicitis, previous EtOH excess, anxiety/depression | 13 | 303 | No | N | N | 38063 | - | - | - | 6761 |
| CV325 | M | White | 32 | Unknown | Gallstone | 2 | 215 | No | N | N | 11235 | - | - | - | - |
| CV328 | F | White | 49 | 4 | Hypertension, hypothyroidism | 11 | 207 | Yes | Y | N | 38863 | - | - | - | - |
| HB01-94 | F | White | 26 | N/A | None | N/A | N/A | N/A | N/A | N/A | 14257 | - | - | - | - |
| HB09-89 | M | White | 31 | N/A | None | N/A | N/A | N/A | N/A | N/A | 5598 | - | - | - | - |
| HB10-82 | F | White | 38 | N/A | None | N/A | N/A | N/A | N/A | N/A | 3276 | - | - | - | - |
| HB72-93 | F | White | 27 | N/A | None | N/A | N/A | N/A | N/A | N/A | 8366 | - | - | - | - |
| HB73-97 | F | White | 23 | N/A | None | N/A | N/A | N/A | N/A | N/A | 6575 | - | - | - | - |
| HB74-76 | M | White | 44 | N/A | None | N/A | N/A | N/A | N/A | N/A | 7055 | - | - | - | - |
| HB75-51 | M | White | 69 | N/A | None | N/A | N/A | N/A | N/A | N/A | 1180 | - | - | - | - |
| HB76-49 | F | White | 71 | N/A | None | N/A | N/A | N/A | N/A | N/A | 9449 | - | - | - | - |
| HB83-44 | F | White | 76 | N/A | None | N/A | N/A | N/A | N/A | N/A | 1748 | - | - | - | - |
| E14 (Ebola) | Unknown | West African | Unknown | N/A | ? | N/A | N/A | N/A | N/A | N/A | 3090 | - | - | - | - |
| E15 (Ebola) | Unknown | West African | Unknown | N/A | ? | N/A | N/A | N/A | N/A | N/A | 3949 | - | - | - | - |
| E2 (Ebola) | Unknown | West African | Unknown | N/A | ? | N/A | N/A | N/A | N/A | N/A | 5243 | - | - | - | - |
| E43 (Ebola) | Unknown | West African | Unknown | N/A | ? | N/A | N/A | N/A | N/A | N/A | 3811 | - | - | - | - |
| E46 (Ebola) | Unknown | West African | Unknown | N/A | ? | N/A | N/A | N/A | N/A | N/A | 2549 | - | - | - | - |
| E50 (Ebola) | Unknown | West African | Unknown | N/A | ? | N/A | N/A | N/A | N/A | N/A | 3114 | - | - | - | - |
| E70 (Ebola) | Unknown | West African | Unknown | N/A | ? | N/A | N/A | N/A | N/A | N/A | 2733 | - | - | - | - |
| E75 (Ebola) | Unknown | West African | Unknown | N/A | ? | N/A | N/A | N/A | N/A | N/A | 3443 | - | - | - | - |
| E82 (Ebola) | Unknown | West African | Unknown | N/A | ? | N/A | N/A | N/A | N/A | N/A | 2835 | - | - | - | - |
| EB215 (Ebola) | M | White | 29 | N/A | ? | N/A | N/A | N/A | N/A | N/A | 8476 | - | - | - | - |
| EB216 (Ebola) | M | White | 34 | N/A | ? | N/A | N/A | N/A | N/A | N/A | 5693 | - | - | - | - |
| EB217 (Ebola) | F | White | 39 | N/A | ? | N/A | N/A | N/A | N/A | N/A | 2861 | - | - | - | - |
| EA003 D0 (RSV Infected) | Unknown | Unknown | Older | N/A | ? | N/A | N/A | N/A | N/A | N/A | 11770 | - | - | - | - |
| EA003 D10 (RSV Infected) | Unknown | Unknown | Older | N/A | ? | N/A | N/A | N/A | N/A | N/A | 3049 | - | - | - | - |
| EA005 D0 (RSV Infected) | Unknown | Unknown | Older | N/A | ? | N/A | N/A | N/A | N/A | N/A | 4510 | - | - | - | - |
| EA005 D10 (RSV Infected) | Unknown | Unknown | Older | N/A | ? | N/A | N/A | N/A | N/A | N/A | 1984 | - | - | - | - |
| EA008 D0 (RSV Infected) | Unknown | Unknown | Older | N/A | ? | N/A | N/A | N/A | N/A | N/A | 10471 | - | - | - | - |
| EA008 D10 (RSV Infected) | Unknown | Unknown | Older | N/A | ? | N/A | N/A | N/A | N/A | N/A | 9503 | - | - | - | - |
| EA014 D0 (RSV Uninfected) | Unknown | Unknown | Older | N/A | ? | N/A | N/A | N/A | N/A | N/A | 7647 | - | - | - | - |
| EA014 D10 (RSV Uninfected) | Unknown | Unknown | Older | N/A | ? | N/A | N/A | N/A | N/A | N/A | 7995 | - | - | - | - |
| EA022 D0 (RSV Uninfected) | Unknown | Unknown | Older | N/A | ? | N/A | N/A | N/A | N/A | N/A | 6213 | - | - | - | - |
| EA022 D10 (RSV Uninfected) | Unknown | Unknown | Older | N/A | ? | N/A | N/A | N/A | N/A | N/A | 8381 | - | - | - | - |
| EA024 D0 (RSV Uninfected) | Unknown | Unknown | Older | N/A | ? | N/A | N/A | N/A | N/A | N/A | 6324 | - | - | - | - |
| EA024 D10 (RSV Uninfected) | Unknown | Unknown | Older | N/A | ? | N/A | N/A | N/A | N/A | N/A | 5135 | - | - | - | - |
| YR001 D0 (RSV Infected) | Unknown | Unknown | Young | N/A | ? | N/A | N/A | N/A | N/A | N/A | 4406 | - | - | - | - |
| YR001 D10 (RSV Infected) | Unknown | Unknown | Young | N/A | ? | N/A | N/A | N/A | N/A | N/A | 6425 | - | - | - | - |
| YR002 D0 (RSV Uninfected) | Unknown | Unknown | Young | N/A | ? | N/A | N/A | N/A | N/A | N/A | 7929 | - | - | - | - |
| YR002 D10 (RSV Uninfected) | Unknown | Unknown | Young | N/A | ? | N/A | N/A | N/A | N/A | N/A | 10690 | - | - | - | - |
| YR003 D0 (RSV Uninfected) | Unknown | Unknown | Young | N/A | ? | N/A | N/A | N/A | N/A | N/A | 3462 | - | - | - | - |
| YR003 D10 (RSV Uninfected) | Unknown | Unknown | Young | N/A | ? | N/A | N/A | N/A | N/A | N/A | 3944 | - | - | - | - |
| YR004 D0 (RSV Infected) | Unknown | Unknown | Young | N/A | ? | N/A | N/A | N/A | N/A | N/A | 8223 | - | - | - | - |
| YR004 D10 (RSV Infected) | Unknown | Unknown | Young | N/A | ? | N/A | N/A | N/A | N/A | N/A | 7751 | - | - | - | - |
| YR006 D0 (RSV Uninfected) | Unknown | Unknown | Young | N/A | ? | N/A | N/A | N/A | N/A | N/A | 4130 | - | - | - | - |
| YR006 D10 (RSV Uninfected) | Unknown | Unknown | Young | N/A | ? | N/A | N/A | N/A | N/A | N/A | 5083 | - | - | - | - |
| YR008 D0 (RSV Infected) | Unknown | Unknown | Young | N/A | ? | N/A | N/A | N/A | N/A | N/A | 15584 | - | - | - | - |
| YR008 D10 (RSV Infected) | Unknown | Unknown | Young | N/A | ? | N/A | N/A | N/A | N/A | N/A | 6257 | - | - | - | - |
| YF193 D0 | M | White | 27 | N/A | None | N/A | N/A | N/A | N/A | N/A | 1192 | - | - | - | - |
| YF199 D0 | M | White | 28 | N/A | None | N/A | N/A | N/A | N/A | N/A | 1243 | - | - | - | - |
| YF207 D0 | F | White | 28 | N/A | None | N/A | N/A | N/A | N/A | N/A | 1039 | - | - | - | - |
| YF193 D28 | M | White | 27 | N/A | None | N/A | N/A | N/A | N/A | N/A | 2175 | - | - | - | - |
| YF199 D28 | M | White | 28 | N/A | None | N/A | N/A | N/A | N/A | N/A | 701 | - | - | - | - |
| YF207 D28 | F | White | 28 | N/A | None | N/A | N/A | N/A | N/A | N/A | 1033 | - | - | - | - |

| Sample | Age | Gender | Ethnicity | Severity Score | Days since symptom onset |
| --- | --- | --- | --- | --- | --- |
| Healthy | 15 ≤50 years old (62.5%)  9 ≥60 years old (37.5%)  (RSV D0s unknown age) | 7 F (29.2%)  5 M (20.8%)  12 Unknown (50%) | 12 White (50%)  12 Unknown (50%) | N/A | N/A |
| COVID-19 | Range: 29-87  Mean: 51.5  Median: 50.5  Mode: N/A  8 ≤50 years old (50%)  5 ≥60 years old (31.25%) | 7 F (43.75%)  9 M (56.25%) | 13 White (81.25%)  1 South East Asian (6.25%)  2 Indian Subcontinent (12.5%) | 4 Unknown (25%)  Range: 1-5  Mean: 2.92  Median: 3  Mode: 3 | Range: 1-35  Mean: 9.6  Median: 8  Mode: 9 |
| COVID-19 Recovered | Range: 28-67  Mean: 49  Median: 50  Mode: N/A | 3 F (60%)  2 M (40%) | 4 White (80%)  1 Indian Subcontinent (20%) | N/A | N/A |
| RSV | **Infected**:  3 Young (50%)  3 Older (50%)  **Uninfected**:  3 Young (50%)  3 Older (50%) | Unknown | Unknown | N/A | N/A |
| Ebola | 3 Young (25%)  9 Unknown (75%) | 1 F (8.3%)  2 M (16.7%)  75% Unknown | 3 White (25%)  9 West African (75%) | N/A | N/A |
| YFV D28 | 3 Young (100%)  Range: 27-28  Mean: 27.7  Median: 28  Mode: 28 | 33.3% F  66.7% M | 3 White (100%) | N/A | N/A |
